# The Global Regulators Lrp, LeuO, and HexA Control Secondary Metabolism in Entomopathogenic Bacteria

**DOI:** 10.3389/fmicb.2017.00209

**Published:** 2017-02-17

**Authors:** Yvonne Engel, Carina Windhorst, Xiaojun Lu, Heidi Goodrich-Blair, Helge B. Bode

**Affiliations:** ^1^Merck-Stiftungsprofessur Molekulare Biotechnologie, Molekulare Biowissenschaften, Goethe Universität FrankfurtFrankfurt am Main, Germany; ^2^Department of Bacteriology, University of Wisconsin–Madison, MadisonWI, USA; ^3^Department of Microbiology, University of Tennessee, Knoxville, KnoxvilleTN, USA; ^4^Buchmann Institute for Molecular Life Sciences (BMLS), Goethe Universität FrankfurtFrankfurt am Main, Germany

**Keywords:** transcription factors, *Photorhabdus*, *Xenorhabdus*, natural product, regulation of natural products

## Abstract

*Photorhabdus luminescens* TTO1 and *Xenorhabdus nematophila* HGB081 are insect pathogenic bacteria and producers of various structurally diverse bioactive natural products. In these entomopathogenic bacteria we investigated the role of the global regulators Lrp, LeuO, and HexA in the production of natural products. Lrp is a general activator of natural product biosynthesis in *X. nematophila* and for most compounds in TTO1. Microarray analysis confirmed these results in *X. nematophila* and enabled the identification of additional biosynthesis gene clusters (BGC) regulated by Lrp. Moreover, when promoters of two *X. nematophila* BGC were analyzed, transcriptional activation by Lrp was observed. In contrast, LeuO in *X. nematophila* and *P. luminescens* has both repressing and activating features, depending on the natural product examined. Furthermore, heterologous overexpression of *leuO* from *X. nematophila* in the closely related *Xenorhabdus szentirmaii* resulted in overproduction of several natural products including novel compounds. The presented findings could be of importance for establishing a tool for overproduction of secondary metabolites and subsequent identification of novel compounds.

## Introduction

**FIGURE 1:**
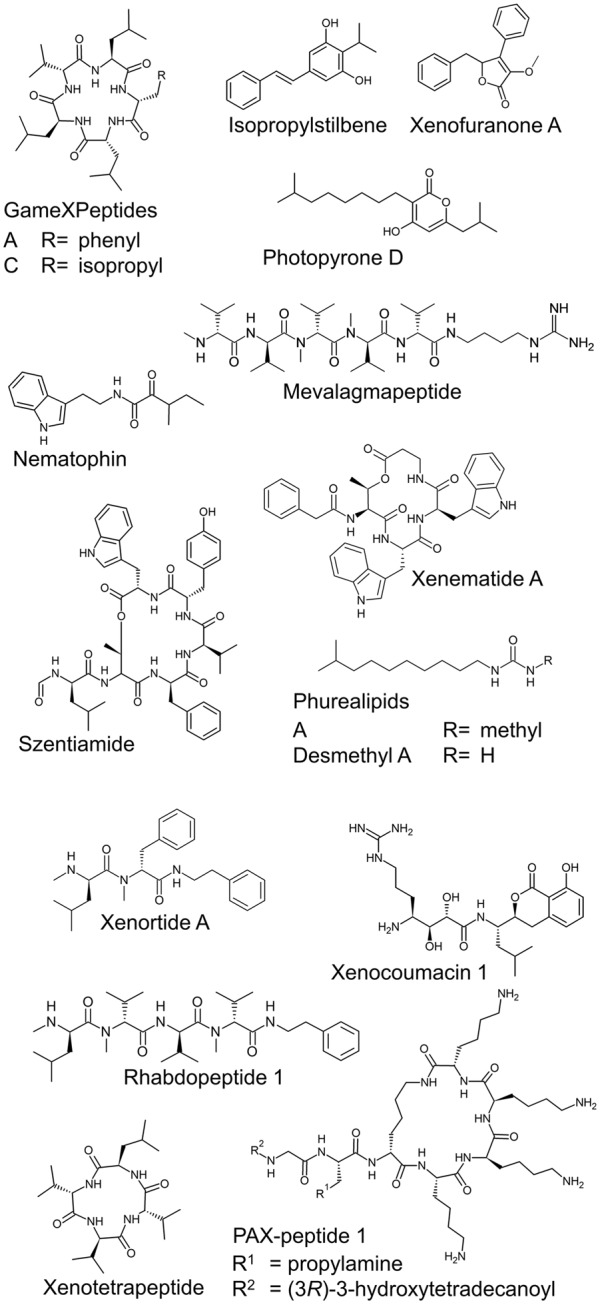
**Examples of natural products from *P. luminescens* TTO1, *X. nematophila* HGB081, and *X. szentirmaii***.

Bacterial natural products are of great importance for our current health system and the development of new therapeutic agents or plant protectants (Zhou et al., 2008; Newman and Cragg, 2012). The entomopathogenic bacterial genera *Photorhabdus* and *Xenorhabdus* are potent producers of structurally diverse compounds (**Figure 1**) that are important during their mutualistic lifestyle in symbiosis with nematodes, the infection of insect larvae and protection of the host cadaver against competitors (Thomas and Poinar, 1979). Within the last few years, advances in understanding regulation in entomopathogenic bacteria have been made, but a general view regarding natural product biosynthesis and function is missing.

Lrp (leucine responsive protein) and LeuO have both been described as global regulators of transcription in *Escherichia coli* (Tani et al., 2002; Shimada et al., 2009) and *Salmonella enterica* (Baek et al., 2009; Dillon et al., 2012). Lrp type regulators are widespread in the bacterial world (Brinkman et al., 2003) and are sensors of several amino acids and thus generally associated with response to nutrient availability (Brinkman et al., 2003; Hart and Blumenthal, 2011). Lrp in *S. enterica* reduces virulence by repressing genes in the *Salmonella* pathogenicity island 1 (SPI-1) and 2 (SPI-2) (Baek et al., 2009) whereas in *Vibrio*, it has been shown to be important for virulence (Lin et al., 2007). In *Xenorhabdus nematophila* Lrp is also a global regulator and affects mutualism with nematodes as well as pathogenicity in insects (Cowles et al., 2007; Hussa et al., 2015). Regarding secondary metabolite production, a *lrp* deletion mutant shows no antibiotic activity toward *Micrococcus luteus* or *Bacillus subtilis* while the wild type has antibiotic activity (Cowles et al., 2007). In *Photorhabdus* an *lrp* mutant has reduced levels of isopropylstilbene (IPS) and its precursor, cinnamic acid. Additionally, Lrp activates *P_stlA_*, the promoter of the gene responsible for cinnamic acid biosynthesis (Lango-Scholey et al., 2013).

LeuO belongs to the largest family of transcriptional regulators in prokaryotes, the LysR type transcriptional regulators (Pareja et al., 2006; Hernández-Lucas and Calva, 2012). In *S. enterica*, where it was first described (Hertzberg et al., 1980; Henikoff et al., 1988) it indirectly acts as a repressor for SPI-1 (Espinosa and Casadesús, 2014) and is often described as an antagonist of heat-stable nucleoid-structuring protein (H-NS) (Chen and Wu, 2005). In *Vibrio cholerae*, however, LeuO is part of the ToxR regulon and down-regulates important virulence factors. The expression of *leuO* in *V. cholerae* is activated by the natural product cyclo(Phe-Pro) (Bina et al., 2013). In *Vibrio parahaemolyticus* a LeuO homolog is positively controlled by ToxRS and negatively regulates transcription of a type III secretion system (Whitaker et al., 2012). As the production of secondary metabolites in *X. nematophila* and *Photorhabdus luminescens* is important for virulence and LeuO is described as a regulator for virulence factors in other bacterial species, we investigated whether LeuO also plays a role in the regulation of natural products in these entomopathogenic bacteria.

HexA is a transcriptional repressor of the LysR type family. In *Photorhabdus temperata* it has an important role in the interaction with the nematode by repressing antibiotic production within the host (Joyce and Clarke, 2003). Kontnik et al. (2010) showed that Δ*hexA* mutants of *P. temperata* and *P. luminescens* produce significantly more IPS and derivatives thereof compared to the wild type while anthraquinone production is upregulated in the *P. temperata* mutant but downregulated in *P. luminescens* Δ*hexA*. Recently it was observed that *hexA* is part of a regulatory cascade in which it is controlled by Hfq. Deletion of *hexA* restores secondary metabolite production in a Δ*hfq* mutant which otherwise shows very little production (Tobias et al., 2016).

In this study, the influence of the three global regulators LeuO, Lrp, and HexA with the focus on secondary metabolite production is described in *P. luminescens* and *X. nematophila*. Furthermore, we examine the potential for heterologous expression of LeuO to modulate natural product production in *Xenorhabdus szentirmaii*.

## Materials and Methods

### Bacterial Strains

The strains used in this work are *E. coli* S17-1 *λpir* (Tp^r^ Sm^r^ *recA* thi *hsdR* RP4-2-Tc::MuKm::Tn7, λ*pir* phage lysogen), *E. coli* DH10B (Life Technologies), *P. luminescens* TTO1 (rifampicin resistant strain) (Duchaud et al., 2003), *X. nematophila* ATCC 19061 (HGB800), *X. nematophila* AN6/1 (HGB081, rifampicin resistant strain, isolated by S. Forst), *X. nematophila lrp-2::*Km (HGB1059) (Cowles et al., 2007), and *X. szentirmaii* DSM 16338 (Lengyel et al., 2005).

### Cultivation of Bacteria

Cells were generally grown in lysogeny broth (LB) with 0.5% (w/v) NaCl or in SF-900 broth (Gibco^TM^) at 30°C or 37°C (for *E. coli*), 200 rpm in Erlenmeyer flasks for liquid cultures. LB plates contained 1.5% agar. Cultures for extraction were inoculated from overnight pre-cultures with a starting OD_600_ of 0.1. For induction of *P_BAD_*, cultures were supplemented with 0.2% (w/v) L-arabinose. Concentrations for antibiotics, added when necessary, were kanamycin (50 μg/mL), rifampicin (50 μg/mL), and chloramphenicol (20 μg/mL). PCR and Sanger DNA sequencing were used to verify mutants.

### Construction of Plasmids

Phusion polymerase (Finnzymes/Thermo Scientific) was used for amplification of PCR products and plasmids were extracted using a Miniprep Kit (Thermo Scientific). Plasmids and purified PCR-products were digested with the described restriction enzymes (Fermentas/Thermo Scientific), purified by gel electrophoresis, extracted with GeneJET^TM^ kit (Fermentas/Thermo Scientific), ligated and transformed into *E. coli* via electroporation.

The pBAD30 plasmid (Guzman et al., 1995) was amplified without *bla* via PCR (oligonucleotides: forward, 5′-CCATGGCATA TATACTTTAG ATTGATTTACG-3′; reverse, 5′-CTCGAGTTCT GCTTAATTTG ATGC-3′) and blunt-end ligated with *P_amp_*-*kanR* (oligonucleotides: forward, 5′-CTCGAGGATA ATAATGGTTT CTTAGACG-3′; reverse, 5′-CCATGGAACT TGGTCTGACA GTTACC-3′) resulting in pBAD30_*kanR* (p15A, *araC*, ara02, and ara01 sites, *P_BAD_, kanR*; details and DNA sequence are depicted in the Supplementary Table S1). Regulator genes *leuO* (TTO1: *plu3672*, 945 bp; HGB081: *XNC1_1043*, 948 bp) and *lrp* (TTO1: *plu1600*, 495 bp; HGB081: *XNC1_1548*, 495 bp) were amplified with overhangs for restriction enzyme sites *Kpn*I and *Pae*I from genomic DNA (extracted with Qiagen Puregene Yeast/Bact. Kit) and subcloned in pJET1.2 (Life Technologies). These restriction enzyme sites were used for ligating the fragments into the corresponding sites of pBAD30_*kanR*. *E. coli* DH10B was transformed with this plasmid and LB kanamycin plates were used to screen for positive clones.

For the construction of cluster expression plasmid (pCEP) plasmids, the start of the genes *leuO, lrp*, and *hexA* from *P. luminescens* TTO1 (oligonucleotides for *leuO*: forward, 5′-**CATATG**GCTG AATACACCTC AGTAACTGC-3′; reverse, 5′-**CTCGAG**GGTA ATGATGAAAAT CCATACG-3′, 547 bp; oligonucleotides for *lrp*: forward, 5′-**CATATG**ATAG ATAATAAAAA ACGTCCGGGA AAAGATC-3′; reverse, 5′-**CTCGAG**CTGG CAAACGCAAC AAAG-3′, 427 bp; oligonucleotides for *hexA*: forward, 5′-**CATATG**TAAA TGCAAATCGT CCGATAATG-3′; reverse, 5′-**CTCGAG**TGCC ATAATACCGG TGTTG-3′, 519 bp) and *X. nematophila* HGB081 (oligonucleotides for *leuO*: forward, 5′-**CATATG**ACTG GATACAACTC GGTAACC-3′; reverse, 5′-**CTCGAG**GGTT ATAACTGATG ACAAACTCTA TTTCC-3′, 517 bp; oligonucleotides for *lrp*: forward, 5′-**CATATG**ATTG ATAATAAGAA GCGTCCAGGA AAAG-3′; reverse, 5′-**CTCGAG**CTCT GTTTTACTTC TTCCATAACA ACATAAGTGC-3′, 476 bp) were amplified from genomic DNA with overhangs for *Nde*I and *Xho*I (bold) and subcloned into pJET1.2 (Life Technologies). After digestion with the corresponding enzymes and clean up (gel electrophoresis and extraction), the fragments were cloned into the corresponding sites of the previously described pCEP vector (Bode et al., 2015). Electrocompetent S17-1 *λpir* cells were transformed with these plasmids. Positive clones (selected on chloramphenicol plates) were then used for conjugation, so that the plasmid could be integrated into *P. luminescens* TTO1 and *X. nematophila* HGB081 by homologous recombination. Selective media containing rifampicin and chloramphenicol were used for identification of positive clones. The resulting four mutant strains, *P. luminescens P_BAD_-leuO* or *P_BAD_-lrp* and *X. nematophila P_BAD_-leuO* or *P_BAD_-lrp* were verified by PCR and sequencing.

To create promoter activity reporters for the gene clusters encoding xenematide (xene) or xenortide (xenor) biosynthesis enzymes, vector pYEYP2.2 (details and DNA sequence are depicted in the Supplementary Table S2) was constructed by exchanging the reporter gene *gfpmut3.1* on pFU69 (Uliczka et al., 2011) with the reporter gene *ypet*, encoding an optimized yellow fluorescent protein (Nguyen and Daugherty, 2005) (Life Technologies). *ypet* as well as the *kanR* resistance cassette were cloned into low copy vector pFU99 (Uliczka et al., 2011), thereby replacing the *lacZ* and *cmR* genes and generating pYEYP2.2 (pSC101^∗^, *kanR, ypet*). Promoter regions *P_Xene_* (oligonucleotides: forward, 5′-ATAT**CTCGAG** CGCCTTACAT CTACAAGCCA-3′ and reverse, 5′-ATAT**GCTAGC** AATAA ATTATACGAA TGTATTCCGT TTACAAG-3′, 446 bp) and *P_Xenor_* (oligonucleotides: forward, 5′-ATAT**CTCGAG** GCTAACAATA GTATGTTAGC ATGGC-3′ and reverse, 5′-ATAT**GCTAGC** ATGGTACTTT TTACCTTTCT GTG-3′, 392 bp) were amplified with overhangs for *Xho*I and *Nhe*I (bold). The corresponding restriction enzyme sites were then used for insertion of the promoter regions into the pYEYP2.2 vector. *E. coli* DH10B was used for these cloning procedures.

### Transformation of *Xenorhabdus* Strains

Promoter reporter and overexpression plasmids were transformed into *X. nematophila* and *X. szentirmaii* via heat shock transformation as described previously (Xu et al., 1989). For this purpose, cultures were grown to OD_600_ ∼0.6 and 1 mL culture was harvested for each individual transformation. Positive clones were isolated on selective media according to antibiotic resistance encoded on the vector.

### Extraction of Natural Products from Bacterial Cultures

For direct extraction of cultures, cells were inoculated with OD_600_ 0.1 in SF-900 broth (Gibco^TM^) (or sorted and inoculated in LB for the FACS analysis) and incubated for 3 days at 30°C, 200 rpm. Subsequently, equal amounts of culture and methanol were mixed. After centrifugation the supernatant was directly analyzed *via* high performance liquid chromatography coupled with mass spectrometry (HPLC-MS).

Cultures for the heterologous *leuO* overexpression in *X. szentirmaii* were inoculated with OD_600_ 0.1 in LB, supplemented with 2% (v/v) Amberlite XAD-16 and harvested after 3 days of cultivation by decanting cells and supernatant. The XAD-16 beads were extracted with methanol with one culture volume for 1 h while stirring. After filtration the extract was evaporated to dryness. For HPLC-MS analysis the extract was redissolved in MeOH in the original culture volume.

### HPLC-MS Analysis

Extracts were analyzed using the UltiMate 3000 LC System (Dionex) coupled to an amaZon X ion trap with electrospray ionization (Bruker Daltonics). A water/acetonitrile gradient with 0.1% formic acid as mobile phase with 0.4 mL/min flow separated the compounds on a C18 column (ACQUITY UPLC BEH, 1.7 mm, Waters). Relative amounts were quantified either directly by integration of the area peak in DataAnalysis 4.2 Software (Bruker Daltonics) or by using Target Analysis (Bruker Daltonics) with the recently described setup (Ahrendt et al., 2015). The *m/z* ratios that were used for this procedure are as follows: *m/z* 215.2 [M+H]^+^ (desmethylphurealipid A), *m/z* 586.4 [M+H]^+^ (GameXPeptide A), *m/z* 552.4 [M+H]^+^ (GameXPeptide C) [M+H]^+^, *m/z* 255.1 [M+H]^+^ (IPS), *m/z* 334.7 [M+2H]^2+^ (mevalagmapeptide), *m/z* 273.2 [M+H]^+^ (nematophin), *m/z* 229.2 [M+H]^+^ (phurealipid A), *m/z* 295.2 [M+H]^+^ (photopyrone D), *m/z* 574.4 [M+H]^+^ (rhabdopeptide 1), *m/z* 838.4 [M+H]^+^ (szentiamide), *m/z* 663.3 [M+H]^+^ (xenematide A), *m/z* 466.3 [M+H]^+^ (xenocoumacin 1), *m/z* 281.1 [M+H]^+^ (xenofuranone A), and *m/z* 410.3 [M+H]^+^ (xenortide A). All analyses were performed in triplicate. Base peak chromatograms and extracted ion chromatograms (EICs) displayed in some figures were also created with DataAnalysis 4.2 (Bruker Daltonics).

### FACS Analysis

Fluorescence activated cell sorting (FACS) analysis and cell sorting were performed on a FACS AriaIII cell sorter (BD Biosciences). YPet was excited with a 488 nm Laser. The filter set up for the YPet channel is 530/30 nm with a 502 LP mirror. At 24 h post-inoculation, 10 μL of culture were diluted with 5 mL phosphate buffered saline (PBS) and one million cells were sorted into 2× LB for each condition to remove dead or inactive cells. These sorted cells were then inoculated into fresh LB with and without arabinose supplementation. Fluorescence analysis was carried out 2 days after this inoculation. For each analysis the culture was diluted 100× with PBS and 50,000 events were recorded with the same instrumental adjustments. At the same time samples for direct HPLC-MS analysis were collected.

### Microarray Analysis

Tiled microarrays were used to measure transcript levels in *X. nematophila* wild type (HGB800) and *lrp-2::*Km early stationary phase cells as previously reported (Hussa et al., 2015) to compare the expression levels of biosynthesis genes. Briefly, the average signal strength of all probes was used as the baseline signal strength level and genes with average signal strength (across probes within that gene) of at least five times this amount were considered expressed. For these genes, the baseline signal strength value was subtracted from the average signal strength for the gene, and all genes were normalized across strains using the values for the *recA* gene. Differences between strains in normalized values of at least twofold were considered differentially expressed. Data shown in **Table 1** represent a subset of genes from this analysis.

**Table 1:** Summary of regulatory effects of Lrp and LeuO on secondary metabolite production in *P. luminescens* and *X. nematophila*.

Compound	Lrp	LeuO	HexA
***Photorhabdus luminescens***			
Mevalagmapeptide	↓	*-*	↓
Photopyrone D	*↑*	*-*	↓
GameXPeptide A	*↑*	*↑*	↓
Isopropylstilbene	*-*	*↑*	↓
Phurealipid A	*↑*	↓	↓
Desmethylphurealipid A	*↑*	↓	↓
***Xenorhabdus nematophila***			
Nematophin	↓	↓	
Xenematide A	*↑*	*↑*	
Xenocoumacin 1	*↑*	↓	
Xenortide A	*↑*	↓*-*	
Rhabdopeptide 1	*↑*	↓	
Xenotetrapeptide	↓	*↑*	

*Downward and upward arrows indicate negative and positive regulation by the regulator, respectively, while hyphen marks no significant change. Arrow and hyphen together show a trend, meaning that a clear difference was observable between wild type and non-induced culture but not between induced and non-induced mutant.*

## Results

### Effects of Lrp, LeuO, and HexA on Selected Natural Products in *Photorhabdus* and *Xenorhabdus*

**FIGURE 2:**
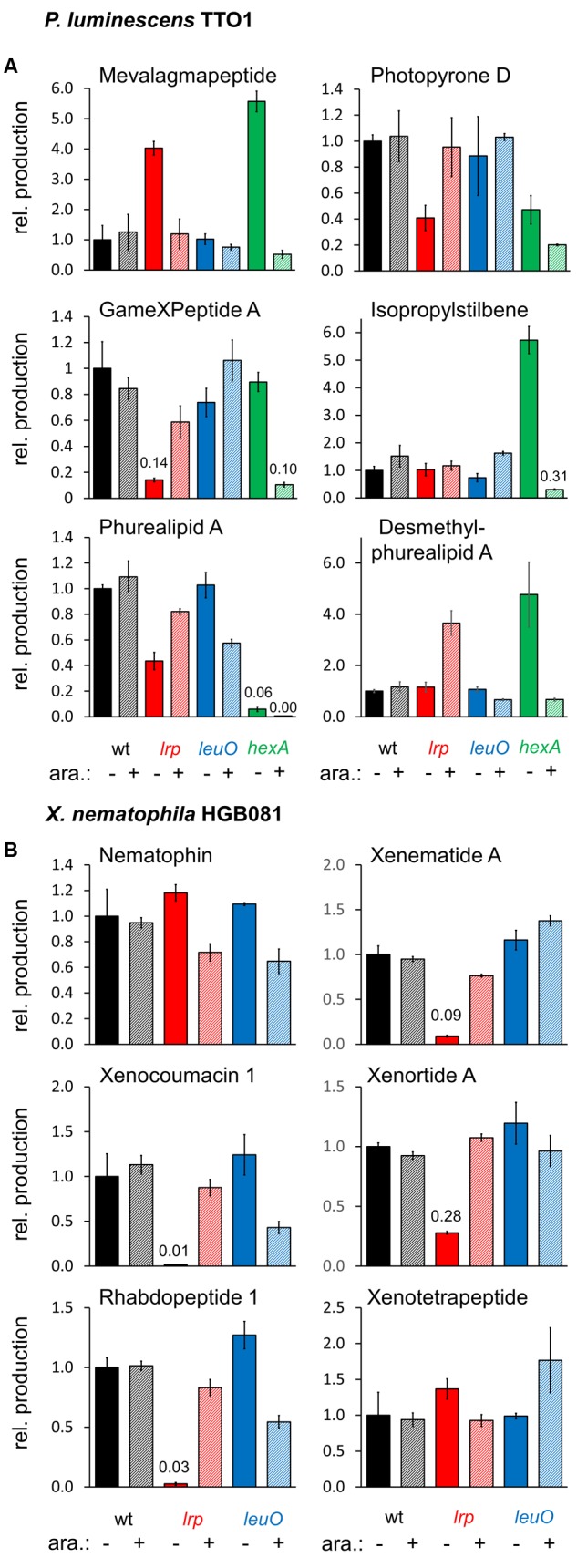
**Relative production of selected natural products in *P. luminescens P_BAD_-lrp* (*lrp*), *P_BAD_-leuO* (*leuO*), and *P_BAD_-hexA* (*hexA*) (A)**, and *X. nematophila* HGB081 *P_BAD_-lrp* (*lrp*) and *P_BAD_-leuO* (*leuO*) **(B)** compared to the respective wild type strains. For induction of *P_BAD_*, cultures were supplemented with 0.2% arabinose (+). Average production of the triplicates (three individual cultures inoculated from the same preculture) was divided by average OD_600_ values and normalized to the appropriate wild type strains without addition of arabinose (**-**). Error bars indicate the standard deviation. Values for compounds produced in very low amounts are presented above the corresponding bars.

To investigate the effects of the global regulators Lrp, LeuO, and HexA on secondary metabolism we created mutants in which the natural promoter of each of these global regulators was exchanged with the inducible *P_BAD_* promoter *via* homologous recombination using the recently described, integrative pCEP vector (Bode et al., 2015). The *P_BAD_* promoter is tightly regulated in *Xenorhabdus* and *Photorhabdus* (Bode et al., 2015), allowing us to control the levels of LeuO or Lrp expressed (either no expression or induced expression) and study downstream regulatory effects. The resulting mutants were grown for 3 days in SF-900 broth, which is a medium for cultivation of insect cell lines and should therefore provide growth conditions that are closer to natural hosts than LB. Extracts were obtained using methanol directly from the cultures and analyzed by HPLC-MS as described in Section “Materials and Methods.” The resulting production was divided by the OD_600_ of the cultures as we observed that some induced and non-induced cultures showed slight differences in their OD_600_ relative to the non-modified wild type cultures (induced: *P. luminescens P_BAD_-lrp*: 0.8×, *P_BAD_-leuO*: 1.1×, *P_BAD_-hexA*: 0.7×, *X. nematophila P_BAD_-lrp*: 1×, *P_BAD_-leuO*: 0.7×; non-induced: *P. luminescens P_BAD_-lrp*: 1.2×, *P_BAD_-leuO*: 0.8×, *P_BAD_-hexA*: 0.6×, *X. nematophila P_BAD_-lrp*: 0.8×, *P_BAD_-leuO*: 1.1×). We found that *P. luminescens* Lrp and LeuO both had either positive or a negative effects on metabolite production, depending on the natural product class while HexA decreased production for all of these compounds (**Figure 2A**).

In the non-induced *P. luminescens P_BAD_-lrp* and *P_BAD_-hexA* strains, we detected four and five times more mevalagmapeptide (Bode et al., 2015) compared to either the wild type or the induced *P_BAD_-lrp* and *P_BAD_-hexA* strains, respectively. Thus, Lrp and HexA act as negative regulators of mevalagmapeptide biosynthesis. In contrast, *leuO* induction had no influence on mevalagmapeptide production. Wild type cultures had higher levels of several compounds including photopyrone D (Nollmann et al., 2015b) and GameXPeptide A (Bode et al., 2012) compared to the non-induced *P_BAD_-lrp* culture, suggesting a role as an activator. However, the induced cultures failed to reach wild type levels (GameXPeptide). The amount of phurealipid A in the induced *P_BAD_-lrp* strain is also lower than in the wild type, however, its precursor desmethylphurealipid A (Nollmann et al., 2015b) is produced by the *P_BAD_-lrp* mutant in higher amounts relative to wild type if *lrp* expression is induced. In summary, the data shows that in *P. luminescens*, Lrp positively regulates production of GameXPeptide A and desmethylphurealipid A, activate photopyrone D and phurealipid A production to a lesser extent, and negatively regulates mevalagmapeptide, but does not significantly affect IPS production.

Based on metabolites produced in the induced versus non-induced cultures of the *P. luminescens P_BAD_-leuO* mutant, LeuO positively influences GameXPeptide A, and IPS production, while it negatively impacts phurealipid A biosynthesis. Desmethylphurealipid A production is hardly influenced by LeuO and the regulation on photopyrone D is not clearly distinguishable. Therefore, *P. luminescens* LeuO acts as both an activator and repressor.

The induced *P_BAD_-hexA* strain shows strongly decreased production of all compounds that were analyzed while the non-induced strain produces higher amounts of mevalagmapeptide (∼5.6×), IPS (∼5.7×), and desmethylphurealipid A (∼4.7×) compared to the wild type while the amounts of photopyrone D and phurealipid A are lower.

In contrast to *P. luminescens* the effects of *lrp* on secondary metabolites in *X. nematophila* are more dramatic: compared to wild type *X. nematophila*, the non-induced *P_BAD_-lrp* mutant strain produces significantly lower levels (∼0.01× to ∼0.28×) of the examined secondary metabolites except for nematophin (Li et al., 1997) and xenotetrapeptide (Kegler et al., 2014). Induced expression of *lrp* restores the production to approximately wild type levels for xenematide A (Lang et al., 2008), xenocoumacin 1 (McInerney et al., 1991), xenortide A (Lang et al., 2008), and rhabdopeptide 1 (Reimer et al., 2013) (**Figure 2B**), indicating that Lrp is predominantly a positive regulator of secondary metabolite production. In contrast, LeuO positively regulates the production of xenotetrapeptide (Kegler et al., 2014) and xenematide A. Compared to non-inducing conditions, the *P_BAD_*_-_*leuO* mutant produced less nematophin (∼0.6×), xenocoumacin 1 (∼0.4×) (McInerney et al., 1991), rhabdopeptide 1 (∼0.5×), and xenortide A (Lang et al., 2008). These data indicate that LeuO can act directly or indirectly as a repressor or activator of *X. nematophila* secondary metabolism.

**FIGURE 3:**
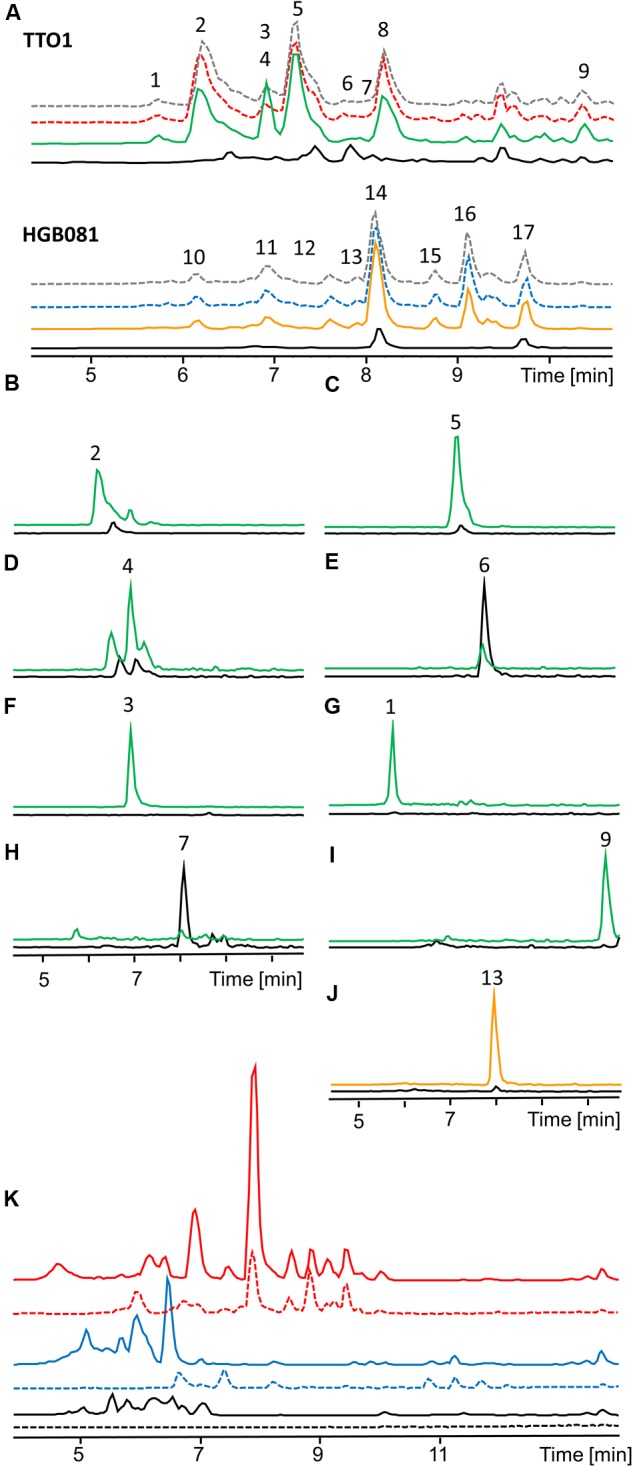
**HPLC-MS analysis of natural product production of *P. luminescens* TTO1 and *X. nematophila* HGB081** (**A**, Base peak chromatogram). Wild type (*P. luminescens*: red dashed, *X. nematophila*: blue dashed), wild type + 0.2% arabinose (gray dashed), *P_BAD_-lrp* (black), *P_BAD_-lrp* + ara (*P. luminescens*: green, *X. nematophila*: orange). (1) *m/z* 379.2 [M+2H]^2+^, (2) *m/z* 312.1 [M+H]^+^, (3) *m/z* 321.1 [M+H]^+^, (4) *m/z* 420.1 [M+H]^+^, (5) *m/z* 326.0 [M+H]^+^, (6) *m/z* 317.0 [M+H]^+^, (7) *m/z* 315.1 [M+H]^+^, (8) anthraquinone, (9) *m/z* 452.1 [M+H]^+^, (10,11) xenocoumacins, (12,14) xenortides, (13) *m/z* 428.2 [M+H]^+^, (15,16) rhabdopeptides, (17) rhabdopeptide and nematophin **(B–J).** Extracted ion chromatograms (EIC) of unidentified regulated compounds in *P_BAD_-lrp* (black), *P_BAD_-lrp* + ara [*P. luminescens* **(B–I)**: green, *X. nematophila* **(J)**: orange]. **(K)** Comparison of base peak chromatograms of *P. luminescens* (blue) and *X. nematophila* (red) wild type cultures grown on LB (continuous line) or SF900 medium (dashed line) and corresponding media controls (black).

The analyses described above provided an opportunity to search for previously not described natural products that might be produced by the various strains we tested. Toward this end we compared the base peak chromatograms of the wild type and the induced and non-induced *P_BAD_*-*lrp* mutants (**Figure 3A**). In several cases, clear differences in peak intensities were observed among strains. We examined the mass spectra at the same retention times to find the *m/z* ratios of the compounds that are responsible for the observed differences. Corresponding EICs for those *m/z* values that do not belong to the already mentioned compounds or their derivatives were generated to display these findings (**Figures 3B–J**). We identified eight new compounds produced by *P. luminescens* (**Figures 3B–I**; *m/z* 312.1 [M+H]^+^, *m/z* 326.0 [M+H]^+^, *m/z* 420.1 [M+H]^+^, *m/z* 317.0 [M+H]^+^, *m/z* 321.1 [M+H]^+^, *m/z* 379.2 [M+2H]^2+^, *m/z* 315.1 [M+H]^+^, *m/z* 452.1 [M+H]^+^). With one exception, each of these individual compounds is present at similar levels in both the wild type and the induced *P_BAD_-lrp* mutant. Only *m/z* 321.1 [M+H]^+^ had a significantly higher production level in the induced *P_BAD_-lrp* mutant than in the wild type. Surprisingly, we also found two compounds that are mainly produced in the non-induced *P_BAD_-lrp* mutant (*m/z* 315.1 [M+H]^+^, *m/z* 317.0 [M+H]^+^). For *X. nematophila* we observed one additional compound (**Figures 3J**; *m/z* 428.2 [M+H]^+^) that is produced in higher amounts in the induced *P_BAD_*-*lrp* mutant culture than in the non-induced culture. All regulatory effects are summarized in **Table 1**.

### Microarray Analysis

A previously reported microarray analysis (Hussa et al., 2015) was mined to examine the influence of Lrp on expression levels of genes encoding (or predicted to encode) secondary metabolite biosynthetic activities. Expression levels of genes within 12 different gene clusters were compared between a *X. nematophila lrp-2*::*kan* mutant (Cowles et al., 2007) and its wild type parent (**Table 2**). Consistent with our metabolite analyses described above, these data indicate that Lrp positively influences the expression of genes involved in xenocoumacin, xenotetrapeptide, rhabdopeptides, xenortides, and xenematides. The genes that encode for PAX-peptide biosynthesis, a compound class that could not be quantified with our HPLC-MS system, were also positively regulated by Lrp. Six additional putative biosynthesis genes or gene clusters of which the biosynthesis product is unknown, also displayed higher levels of expression in wild type relative to the *lrp* mutant. Only one putative biosynthesis gene (*XNC1_2799*) displayed negative regulation by Lrp. Overall, the microarray results provide evidence that Lrp influences secondary metabolite production at the level of gene expression (transcription or RNA stability).

**Table 2:** Fold differences in gene expression levels of *X. nematophila* HGB800 wild type (wt) versus *lrp-2::kan* (HGB1059).

Natural product	ID	Annotation	wt/lrp
Unknown	*XNC1_0646*	Non-ribosomal peptide synthetase (NRPS, fragment)	13.75
Unknown	*XNC1_1561*	NRPS	14.98
Xenocoumacins	*XNC1_1698*	Desaturase XcnN	3.43
	*XNC1_1699*	Dehydrogenase XcnM	8.00
	*XNC1_1700*	Polyketide synthase (PKS) XcnL	11.15
	*XNC1_1701*	Non-ribosomal peptide synthase XcnK	12.11
	*XNC1_1702*	Conserved hypothetical protein XcnJ	9.62
	*XNC1_1703*	Thioesterase XcnI	11.81
	*XNC1_1704*	PKS XcnH	13.41
	*XNC1_1705*	Peptidase XcnG	11.04
	*XNC1_1706*	PKS XcnF	12.05
	*XNC1_1707*	Acyl-CoA dehydrogenase XcnE	15.66
	*XNC1_1708*	Putative acyl carrier protein XcnD	14.14
	*XNC1_1709*	Methoxymalonate biosynthesis protein XcnC	18.31
	*XNC1_1710*	3-hydroxyacyl-CoA dehydrogenase XcnB	17.77
	*XNC1_1711*	NRPS XcnA	12.43
Xenotetrapeptide	*XNC1_2022*	NRPS XtpS	2.14
Unknown	*XNC1_2038*	NRPS	2.15
	*XNC1_2039*	NRPS	2.68
	*XNC1_2040*	NRPS	2.11
Unknown	*XNC1_2152*	Hypothetical protein	3.67
	*XNC1_2153*	Arginine aminomutase	29.52
	*XNC1_2154*	Aminotransferase	16.99
	*XNC1_2155*	Putative clavaminate synthase	11.40
	*XNC1_2156*	PKS	9.17
	*XNC1_2157*	PKS/NRPS hybrid	5.75
	*XNC1_2158*	Putative membrane protein	5.41
	*XNC1_2159*	Putative 2-dehydropantoate 2-reductase	4.05
	*XNC1_2160*	Putative dioxygenase	2.88
	*XNC1_2161*	NRPS	3.04
	*XNC1_2162*	Thioesterase (fragment)	2.47
Rhabdopeptides	*XNC1_2228*	NRPS RdpA	6.37
	*XNC1_2229*	NRPS RdpB	8.50
	*XNC1_2230*	NRPS RdpC	7.46
Unknown	*XNC1_2233*	NRPS	2.03
Xenortides	*XNC1_2299*	NRPS XndA	3.57
	*XNC1_2300*	NRPS XndB	4.50
Unknown	*XNC1_2464*	NRPS	1.98
	*XNC1_2465*	NRPS	2.27
	*XNC1_2466*	NRPS	2.61
	*XNC1_2467*	NRPS	2.89
	*XNC1_2468*	Putative *N*-acetyltransferase	3.99
	*XNC1_2469*	Putative hydroxylase	5.06
	*XNC1_2470*	Putative aminotransferase	5.67
Xenematides	*XNC1_2713*	NRPS	5.33
PAX-Peptides	*XNC1_2781*	NRPS XpsB	8.91
	*XNC1_2782*	NRPS XpsB	9.93
	*XNC1_2783*	NRPS XpsA	10.14
	*XNC1_2784*	Putative ABC transporter	10.10
Unknown	*XNC1_2799*	NRPS (fragment)	0.36

### Lrp Influences Promoter Activity

**FIGURE 4:**
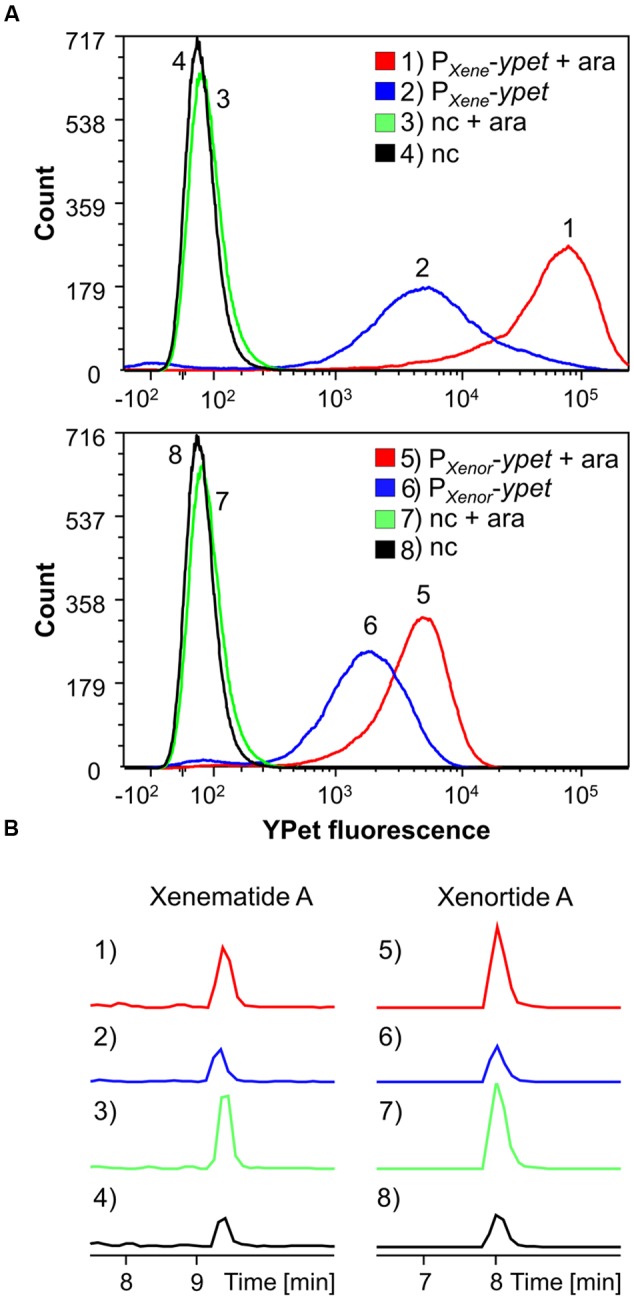
**FACS analysis detecting YPet (yellow fluorescent protein) fluorescence intensity (per individual cell) of 50,000 events for each condition (A)** and HPLC-MS analysis **(B)** detecting xenematide A (1–4) or xenortide A (5–8). Analyses were performed on *X. nematophila* HGB081 *P_BAD_-lrp* carrying *ypet* promoter-reporter plasmids for *P_Xene_* (1, 2) or *P_Xenor_* (5, 6) or an empty vector negative control (nc; 3, 4, 7, 8). Strains were either induced (+ara) or not induced by addition of 0.2% arabinose to growth cultures. (1–4) EIC *m/z* 466.3 [M+H]^+^ for xenematide A. (5–8) EIC *m/z* 410.3 [M+H]^+^ for xenortide A. **Figure 4A** was created with FCS Express 5.

To further explore the influence of Lrp on transcription of secondary metabolite gene clusters, we created reporter constructs that placed the yellow-fluorescent-protein YPet under control of the promoters driving expression of Lrp-dependent xenematide A (xene) and xenortide A (xenor) biosynthesis genes: *P_Xene_-ypet* and *P_Xenor_*-*ypet* reporters, respectively. These reporters and a negative control (nc, promoterless reporter plasmid) were expressed in *X. nematophila P_BAD_*-*lrp* from low-copy plasmids (pYEYP2.2) and analyzed by FACS-analysis under non-inducing and inducing growth conditions. Increased promoter activity, based on YPet fluorescence, was observed on single cell level when *lrp* was induced with arabinose for *P_Xene_* and *P_Xenor_*, suggesting an effect on transcriptional regulation of the corresponding biosynthesis genes by Lrp (**Figure 4A**). Extracts of the same cultures supports these findings as the induced cultures contain a higher level of the corresponding compounds (**Figure 4B**).

### Effects of Heterologous Expression of *leuO* on Metabolite Production

**FIGURE 5:**
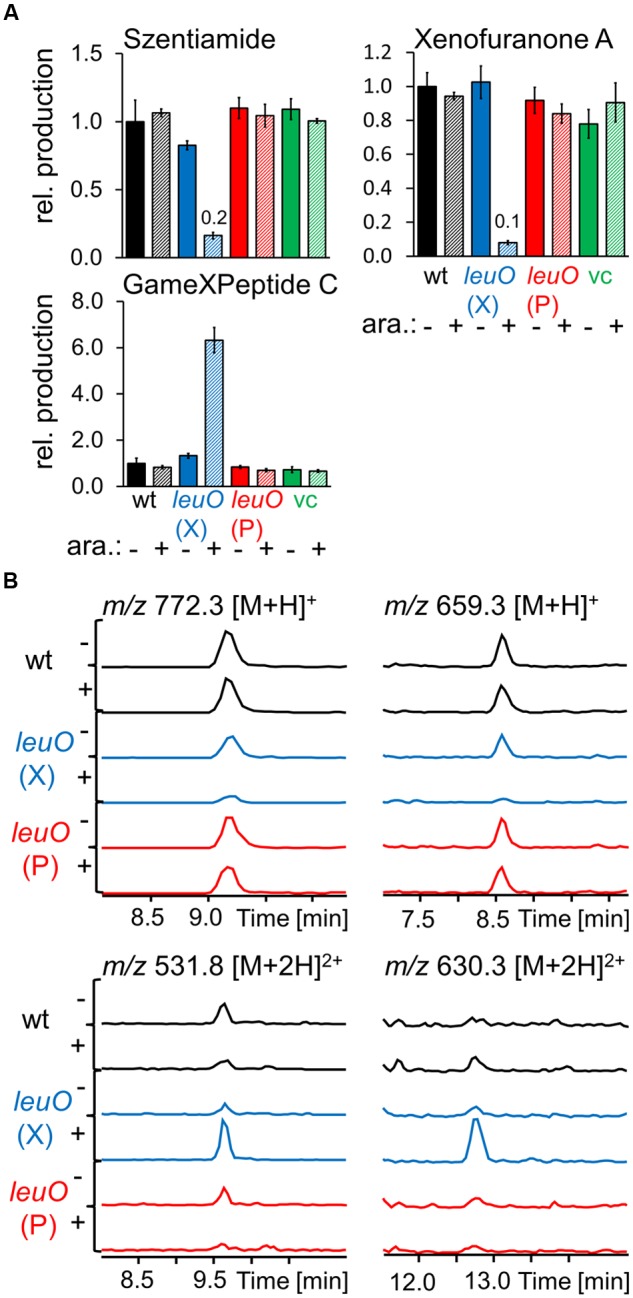
**Production of selected natural products after heterologous expression of *leuO* from *P. luminescens* TTO1 (P) and *X. nematophila* HGB081 (X) in *X. szentirmaii* compared to wild type and vector control (vc) as analyzed by HPLC-MS.** Cultures supplemented with 0.2% arabinose for *P_BAD_* induction are striped and marked with (+) otherwise with (-). **(A)** Average production of the triplicates (three individual cultures inoculated from the same preculture) was normalized to the appropriate wild type strains without addition of arabinose (-), error bars mark the standard deviation. Values for compounds produced in very low amounts are presented above the corresponding bars. **(B)** EICs of regulated but structurally unknown compounds.

Based on our finding that induced expression of *lrp* and *leuO* could reveal previously unidentified compounds, we considered the possibility that their ectopic expression in a heterologous host might allow expanded natural product discovery. To test if LeuO could function to control secondary metabolite production in a heterologous host, we generated pBAD30_*kanR*-based arabinose-inducible overexpression plasmids pBAD30_*kanR*_*leuO*_P encoding LeuO from *P. luminescens* and pBAD30_*kanR*_*leuO*_X encoding LeuO from *X. nematophila*. When these were expressed in *X. szentirmaii* we found that induced expression of *X. nematophila leuO* had major effects on secondary metabolite production while *P. luminescens leuO* induction did not visibly alter production of tested compounds (**Figure 5**). As in the endogenous *X. nematophila* strain, it had both positive and negative consequences on compound production in *X. szentirmaii*. Production of GameXPeptide C, the main GameXPeptide derivative in *X. szentirmaii*, is increased sixfold while xenofuranone (Brachmann et al., 2006) and szentiamide (Ohlendorf et al., 2011) production were reduced to ∼0.1× and ∼0.2× compared to the wild type, respectively (**Figure 5**). By comparison of the induced and non-induced base peak chromatograms new compounds with *m/z* 659.3 and 772.3 [M+H]^+^ were identified (**Figure 5**) that might belong to the family of rhabdopeptides due to their characteristic fragmentation pattern [Reimer et al., 2013; see Supporting Information (Supplementary Figure S1)] as well as *m/z* 531.3 [M+2H]^2+^ and *m/z* 630.3 [M+2H]^2+^ which are structurally still unknown. High resolution high performance liquid chromatography coupled with mass spectrometry (HR-HPLC-MS) suggests a sum formula of C_52_H_92_N_11_O_12_ for *m/z* 531.3 [M+2H]^2+^ indicating a peptide structure.

## Discussion

### The Role of Lrp, LeuO, and HexA on Natural Product Biosynthesis in Entomopathogenic Bacteria

Understanding the regulation of natural product biosynthesis is an important step toward predicting the function of natural products if it is known under which conditions the regulators are active. The regulatory cascades controlling natural product biosynthesis have not been investigated in detail in entomopathogenic bacteria. The global regulator HexA has already been the focus of several studies on *Photorhabdus* and was found to play an important role as a repressor in the nematode host. It has been reported that it has an impact on IPS and anthraquinone biosynthesis (Kontnik et al., 2010) and that HexA itself is regulated by another global regulator. A Δ*hfq* mutant hardly produces secondary metabolites while an additional deletion of *hexA* restores the production (Tobias et al., 2016). We wanted to enhance these studies with the focus on additional compounds directly comparing *hexA* inducing and non-inducing conditions. Indeed we observed that HexA generally acts as a repressor for all compounds that have been analyzed in *P. luminescens*. Lrp as a global transcriptional regulator has been reported in both *Photorhabdus* and *Xenorhabdus*, but not directly upon natural product BGC (Cowles et al., 2007; Kontnik et al., 2010; Lango-Scholey et al., 2013; Hussa et al., 2015). The greatest influence of *P. luminescens* TTO1 Lrp on a known compound is the overproduction of desmethylphurealipid A (∼4×) while LeuO has very little effect (**Figure 2**). Overproduction (5×) for this compound was also observed in the non-induced *P_BAD_-hexA* strain. *In vivo* experiments in which this compound and other phurealipids were injected into *Galleria mellonella* and *Manduca sexta* larvae showed reduced levels of mRNA for insect antimicrobial-peptide-encoding genes suggesting a role for these molecules in insect pathogenicity (Nollmann et al., 2015b). In contrast, the non-induced Lrp and HexA mutant both produce significantly more mevalagmapeptide than the wild type (∼4× and ∼5×, respectively) or the induced mutant. The role of mevalagmapeptides for the producing organism has yet to be established. Lrp has previously been reported to have either no significant (Kontnik et al., 2010), as well as activating effects (Lango-Scholey et al., 2013), on IPS biosynthesis. Lango-Scholey et al. (2013) suggested that this discrepancy might be due to small differences in the corresponding lab strains. In our study, we found little influence of Lrp on stilbene production. However, we saw activation by LeuO on its biosynthesis, while HexA represses it. If *hexA* is not induced, IPS levels are ∼6× above wild type level, which supports previous studies (Kontnik et al., 2010). We suggest that other factors such as nutrients might play an additional role for Lrp in regulating IPS biosynthesis, as we also observed an increase in IPS production for the induced strain when LB was used for cultivation instead of SF-900 broth (data not shown). For the decreased IPS production in the absence of Lrp observed by Lango-Scholey et al. (2013), the limiting factor appeared to be the branched-chain α-ketoacid dehydrogenase encoded in the *bkdABC* operon that is part of the IPS biosynthesis pathway (Joyce et al., 2008) and is also responsible for the production of branched chain fatty acids (BCFAs) in *P. luminescens* (Lango-Scholey et al., 2013). BCFAs are also part of the photopyrone and phurealipid biosynthesis and their production is also negatively affected by the absence of Lrp. The photopyrones are signal molecules inducing production of the *Photorhabdus* clumping factor (*pcf*) through a recently described cell–cell communication system (Brachmann et al., 2013). Heterologous expression of the *pcf* operon in *E. coli* greatly enhanced the toxicity toward insect larvae, demonstrating the contribution of photopyrones toward the virulence of *P. luminescens*.

Except for nematophin and xenotetrapeptide, Lrp strongly activates secondary metabolite production in *X. nematophila*, while LeuO attenuates the production of most examined natural products, including nematophin. Nematophin possesses antifungal and antibacterial activities (Li et al., 1997) that might be restricted to *Staphylococcus* strains (Kennedy et al., 2000) although Lang et al. (2008) could neither reproduce these effects, nor could they determine an activity against other bacterial species. Xenocoumacins, xenematides, and xenortides display antimicrobial activity against a broad spectrum of bacteria (Lang et al., 2008) and are thought to eliminate microbial competitors to defend the nutrients within the insect cadaver. Xenematides have insecticidal activity, rhabdopeptides are active against insect hemocytes (Lang et al., 2008; Reimer et al., 2013), and xenortides are cytotoxic against mammalian L6 cells (Reimer et al., 2014). The regulation of these compounds by Lrp is in agreement with the previously reported finding that this regulator is needed for virulence (Cowles et al., 2007). Additionally, we show that Lrp affects promoter activity for *P_Xene_* and *P_Xenor_* (**Figure 4A**). Consistent with these data, microarray analysis revealed Lrp-dependent regulation of the known biosynthesis genes and clusters for xenematides, xenortides, rhabdopeptides, xenocoumacins, and PAX-peptides, as well as six other biosynthetic genes of which the exact function is as yet unknown. Each of these clusters was identified through RNAseq analysis as being positively regulated by the global regulator FliZ in the F1 strain of *X. nematophila* (Jubelin et al., 2013). Since Lrp also positively regulates *fliZ* the likely regulatory hierarchy is one in which Lrp positively regulates FliZ, which in turn activates expression of biosynthetic clusters (Hussa et al., 2015). However, members of the Lrp regulon that are either directly or indirectly regulated by Lrp remain to be distinguished. The microarray analysis also shows a slightly higher gene expression for the xenotetrapeptide biosynthesis gene although the relative quantification of this compound revealed a slight downregulation by Lrp. This discrepancy may be explained by differences in growth conditions (LB versus SF-900) or strain identities (ATCC19061 versus AN6/1). One biosynthetic gene, *XNC1_2799* is apparently negatively regulated by Lrp. LeuO in *X. nematophila* acts as a repressor for all examined secondary metabolites except xenotetrapeptide and xenematide A, analogous to its role in *V. cholerae, V. parahaemolyticus*, and *S. enterica* in which this regulator attenuates virulence (Whitaker et al., 2012; Bina et al., 2013; Espinosa and Casadesús, 2014). This is in contrast to *P. luminescens* TTO1 where LeuO acts as an activator as well as a repressor of secondary metabolism.

As we found that the non-induced *P_BAD_-lrp* mutants produced a significantly lower amount of several compounds but the induced expression of Lrp did not fully restore the production, it is likely that the induced constitutive expression does not reach the natural Lrp levels.

### Heterologous Expression of *leuO* Reveals Control on Secondary Metabolism in *X. szentirmaii*

Surprisingly, we found that expression of *X. nematophila leuO* in *X. szentirmaii* has a major influence on secondary metabolite expression while *P. luminescence leuO* has no effects (**Figure 5**). The *X. nematophila* and *X. szentirmaii* LeuO sequences are more similar to each other (90% based on blastx analysis) than either is to *P. luminescens* LeuO (71 and 71%, respectively) indicating that the *P. luminescens* regulator may not be functional in *Xenorhabdus* strains. The impact of LeuO on the synthesis of two natural products is particularly notable when comparing these three organisms. In *P. luminescens* and *X. szentirmaii* GameXPeptides are positively regulated by *leuO* expression. Similarly, rhabdopeptide biosynthesis in *X. nematophila* and *X. szentirmaii* is attenuated by LeuO. The finding that the global regulator LeuO controls the production of the same compound classes indicates that, to some extent, it plays a similar role in the different entomopathogenic *Photorhabdus* and *Xenorhabdus* species.

### Global Regulators as Tools for the Discovery of New Secondary Metabolites and Increased Production of Useful Molecules

The development of new drugs, especially new antibiotics, is an important research area as resistances against common antibiotics are increasing dramatically (WHO, 24 July 2015). ^1^ The entomopathogenic *Photorhabdus* and *Xenorhabdus* spp. are a rich source of compounds with antimicrobial bioactivity, but isolation for structural characterization and bioactivity assays is often problematic as the amounts are often low. One method for improving biosynthesis is the exchange of the promoter in front of the biosynthesis gene (Bode et al., 2015) or heterologous expression (Schimming et al., 2014). Unfortunately, these approaches require knowledge about the biosynthetic pathways and the genes necessary for their expression. In the case of heterologous expression another issue is that some biosynthetic precursors might be missing in the heterologous host organism (Nollmann et al., 2015a). One approach that avoids these issues is to elicit overproduction of the molecule(s) in the original producers without knowledge of the relevant biological pathways. Based on the work presented here, the global regulators Lrp and LeuO could be exploited for this approach. For example, overproduction could be achieved by expression of regulators under control of a strong constitutive promoter, or the use of a replicative overexpression plasmid. The potential success of this strategy is supported by our finding that ectopic expression of the *X. nematophila* LeuO regulator in *X. szentirmaii* led to overproduction of the GameXPeptide (**Figure 5**). Further, this approach can also reveal previously unrecognized compounds, such as the two compounds with *m/z* 531.3 [M+2H]^2+^ and *m/z* 630.3 [M+2H]^2+^ expressed by *X. szentirmaii* carrying the *X. nematophila* LeuO. The heterologous overexpression of activators like LeuO or Lrp from diverse *Photorhabdus* and *Xenorhabdus* strains offers the possibility to increase the production of specific compounds in strains that have not been sequenced yet.

Although requiring *a priori* knowledge of genome sequence, the inducible production of Lrp in endogenous hosts also has the power to reveal novel compounds. For instance, by comparing base peak chromatograms and the corresponding mass spectra, we identified nine new compounds produced by *P. luminescens* and *X. nematophila*. This discovery might mainly be due to the use of SF-900 broth, a medium that is used for culturing insect cell lines instead of the normally used LB, as most compounds were also present in the wild type and the production levels between wild type and the induced mutant were similar for the individual compounds. Three of these natural products were produced in significantly higher amounts in the induced (*m/z* 321.1 [M+H]^+^) or non-induced (*m/z* 315.0 [M+H]^+^, *m/z* 317.0 [M+H]^+^) *P. luminescens P_BAD_*-*lrp* mutant than in the wild type, suggesting the capacity of this approach to expand the discernible array of secondary metabolites. For comparison of production in SF900 and LB, the base peak chromatograms of *P. luminescens* and *X. nematophila* extracts are displayed in **Figures 3K**. It is visible that components of LB disturb the chromatogram between retention time (RT) 5–7 min while it is easier to observe new compounds in the cleaner background of SF900 medium.

## Conclusion

We have established that the global regulators Lrp, HexA, and LeuO are involved in the regulation of secondary metabolism in *X. nematophila* and *P. luminescens*, with different effects (activating, repressing, or both) depending on the organism and the individual natural products. A potential for increasing secondary metabolites in heterologous hosts has been shown for *X. nematophila* LeuO in *X. szentirmaii*. The heterologous expression of *lrp* and *leuO* in other organisms remains to be studied but promises to reveal previously unrecognized metabolites.

## Author Contributions

YE performed all experiments except the microarray analysis, which were performed by XL and analyzed by XL and HG-B. The *P. luminescens* and *X. nematophila* promoter exchange mutants and the *X. szentirmaii* pBAD30_kanR strains were performed by CW. HB and YE wrote the paper with input from all authors.

## Conflict of Interest Statement

The authors declare that the research was conducted in the absence of any commercial or financial relationships that could be construed as a potential conflict of interest.

## Footnotes

1 www.who.int/mediacentre/factsheets/fs194/en/

## References

[B1] Ahrendt T. Wolff H. Bode H. B. (2015). The lipidome of neutral and phospholipids of *Myxococcus xanthus* during fruiting body formation and germination. *Appl. Environ. Microbiol.* 2015:01537-15 10.1128/AEM.01537-15 PMC4561683 26162876

[B2] Baek C.-H. Wang S. Roland K. L. Curtiss R. (2009). Leucine-responsive regulatory protein (Lrp) acts as a virulence repressor in *Salmonella enterica* serovar Typhimurium. *J. Bacteriol.* 191 1278–1292. 10.1128/JB.01142-08 19074398 PMC2631999

[B3] Bina X. R. Taylor D. L. Vikram A. Ante V. M. Bina J. E. (2013). *Vibrio cholerae* ToxR downregulates virulence factor production in response to cyclo(Phe-Pro). *mBio* 4:e366-13 10.1128/mBio.00366-13 PMC3760244 23982069

[B4] Bode E. Brachmann A. O. Kegler C. Simsek R. Dauth C. Zhou Q. (2015). Simple “on-demand” production of bioactive natural products. *Chembiochem* 16 1115–1119. 10.1002/cbic.201500094 25826784

[B5] Bode H. B. Reimer D. Fuchs S. W. Kirchner F. Dauth C. Kegler C. (2012). Determination of the absolute configuration of peptide natural products by using stable isotope labeling and mass spectrometry. *Chem. Eur. J.* 18 2342–2348. 10.1002/chem.201103479 22266804

[B6] Brachmann A. O. Brameyer S. Kresovic D. Hitkova I. Kopp Y. Manske C. (2013). Pyrones as bacterial signaling molecules. *Nat. Chem. Biol.* 9 573–578. 10.1038/nCHeMBIO.1295 23851573

[B7] Brachmann A. O. Forst S. Furgani G. M. Fodor A. Bode H. B. (2006). Xenofuranones A and B: phenylpyruvate dimers from *Xenorhabdus szentirmaii*. *J. Nat. Prod.* 69 1830–1832. 10.1021/np060409n 17190473

[B8] Brinkman A. B. Etterna T. J. de Vos W. M. Oost J. van der (2003). The Lrp family of transcriptional regulators. *Mol. Microbiol.* 48 287–294. 10.1046/j.1365-2958.2003.03442.x 12675791

[B9] Chen C.-C. Wu H.-Y. (2005). LeuO protein delimits the transcriptionally active and repressive domains on the bacterial chromosome. *J. Biol. Chem.* 280 15111–15121. 10.1074/jbc.M414544200 15711009

[B10] Cowles K. N. Cowles C. E. Richards G. R. Martens E. C. Goodrich-Blair H. (2007). The global regulator Lrp contributes to mutualism, pathogenesis and phenotypic variation in the bacterium *Xenorhabdus nematophila*. *Cell Microbiol.* 9 1311–1323. 10.1111/j.1462-5822.2006.00873.x 17223926

[B11] Dillon S. C. Espinosa E. Hokamp K. Ussery D. W. Casadesús J. Dorman C. J. (2012). LeuO is a global regulator of gene expression in *Salmonella enterica* serovar Typhimurium. *Mol. Microbiol.* 85 1072–1089. 10.1111/j.1365-2958.2012.08162.x 22804842

[B12] Duchaud E. Rusniok C. Frangeul L. Buchrieser C. Givaudan A. Taourit S. (2003). The genome sequence of the entomopathogenic bacterium *Photorhabdus luminescens*. *Nat. Biotechnol.* 21 1307–1313. 10.1038/nbt886 14528314

[B13] Espinosa E. Casadesús J. (2014). Regulation of *Salmonella enterica* pathogenicity island 1 (SPI-1) by the LysR-type regulator LeuO. *Mol. Microbiol.* 91 1057–1069. 10.1111/mmi.12500 24354910

[B14] Guzman L. M. Belin D. Carson M. J. Beckwith J. (1995). Tight regulation, modulation, and high-level expression by vectors containing the arabinose PBAD promoter. *J. Bacteriol.* 177 4121–4130. 10.1128/jb.177.14.4121-4130.1995 7608087 PMC177145

[B15] Hart B. R. Blumenthal R. M. (2011). Unexpected coregulator range for the global regulator Lrp of *Escherichia coli* and *Proteus mirabilis*. *J. Bacteriol.* 193 1054–1064. 10.1128/JB.01183-10 21169483 PMC3067584

[B16] Henikoff S. Haughn G. W. Calvo J. M. Wallace J. C. (1988). A large family of bacterial activator proteins. *Proc. Natl. Acad. Sci. U.S.A.* 85 6602–6606. 10.1073/pnas.85.18.6602 3413113 PMC282025

[B17] Hernández-Lucas I. Calva E. (2012). The coming of age of the LeuO regulator. *Mol. Microbiol.* 85 1026–1028. 10.1111/j.1365-2958.2012.08175.x 22812455

[B18] Hertzberg K. M. Gemmill R. Jones J. Calvo J. M. (1980). Cloning of an EcoRI-Generated fragment of the leucine operon of *Salmonella* typhimurium. *Gene* 8 135–152. 10.1016/0378-1119(80)90033-5 6987127

[B19] Hussa E. A. Casanova-Torres ÁM. Goodrich-Blair H. (2015). The global transcription factor Lrp controls virulence modulation in *Xenorhabdus nematophila*. *J. Bacteriol.* 197 3015–3025. 10.1128/JB.00272-15 26170407 PMC4542165

[B20] Joyce S. A. Brachmann A. O. Glazer I. Lango L. Schwär G. Clarke D. J. (2008). Bacterial biosynthesis of a multipotent stilbene. *Angew. Chem. Int. Ed.* 47 1942–1945. 10.1002/anie.200705148 18236486

[B21] Joyce S. A. Clarke D. J. (2003). A hexA homologue from *Photorhabdus* regulates pathogenicity, symbiosis and phenotypic variation. *Mol. Microbiol.* 47 1445–1457. 10.1046/j.1365-2958.2003.03389.x 12603747

[B22] Jubelin G. Lanois A. Severac D. Rialle S. Longin C. Gaudriault S. (2013). FliZ is a global regulatory protein affecting the expression of flagellar and virulence genes in individual *Xenorhabdus nematophila* bacterial cells. *PLoS Genet.* 9:e1003915 10.1371/journal.pgen.1003915 PMC3814329 24204316

[B23] Kegler C. Nollmann F. I. Ahrendt T. Fleischhacker F. Bode E. Bode H. B. (2014). Rapid determination of the amino acid configuration of xenotetrapeptide. *Chembiochem* 15 826–828. 10.1002/cbic.201300602 24616055

[B24] Kennedy G. Viziano M. Winders J. A. Cavallini P. Gevi M. Micheli F. (2000). Studies on the novel anti-staphyloccal compound nematophin. *Bioorg. Med. Chem. Lett.* 10 1751–1754. 10.1016/S0960-894X(00)00331-0 10937740

[B25] Kontnik R. Crawford J. M. Clardy J. (2010). Exploiting a global regulator for small molecule discovery in *Photorhabdus luminescens*. *ACS Chem. Biol.* 5 659–665. 10.1021/cb100117k 20524642 PMC2912427

[B26] Lang G. Kalvelage T. Peters A. Wiese J. Imhoff J. F. (2008). Linear and cyclic peptides from the entomopathogenic bacterium *Xenorhabdus nematophilus*. *J. Nat. Prod.* 71 1074–1077. 10.1021/np800053n 18491867

[B27] Lango-Scholey L. Brachmann A. O. Bode H. B. Clarke D. J. (2013). The expression of stlA in *Photorhabdus luminescens* is controlled by nutrient limitation. *PLoS ONE* 8:e82152 10.1371/journal.pone.0082152 PMC3838401 24278476

[B28] Lengyel K. Lang E. Fodor A. Szállás E. Schumann P. Stackebrandt E. (2005). Description of four novel species of *Xenorhabdus*, family Enterobacteriaceae: *Xenorhabdus budapestensis* sp. nov., *Xenorhabdus ehlersii* sp. nov., *Xenorhabdus innexi* sp. nov., and *Xenorhabdus szentirmaii* sp. nov. *Syst. Appl. Microbiol.* 28 115–122. 10.1016/j.syapm.2004.10.004 15830803

[B29] Li J. Chen G. Webster J. M. (1997). Nematophin, a novel antimicrobial substance produced by *Xenorhabdus nematophilus* (Enterobactereaceae). *Can. J. Microbiol.* 43 770–773. 10.1139/m97-110 9304787

[B30] Lin W. Kovacikova G. Skorupski K. (2007). The quorum sensing regulator HapR downregulates the expression of the virulence gene transcription factor AphA in *Vibrio cholerae* by antagonizing Lrp- and VpsR-mediated activation. *Mol. Microbiol.* 64 953–967. 10.1111/j.1365-2958.2007.05693.x 17501920

[B31] McInerney B. V. Taylor W. C. Lacey M. J. Akhurst R. J. Gregson R. P. (1991). Biologically Active Metabolites from *Xenorhabdus* Spp., Part 2. Benzopyran-1-one derivatives with gastroprotective activity. *J. Nat. Prod.* 54 785–795. 10.1021/np50075a006 1955881

[B32] Newman D. J. Cragg G. M. (2012). Natural products as sources of new drugs over the 30 years from 1981 to 2010. *J. Nat. Prod.* 75 311–335. 10.1021/np200906s 22316239 PMC3721181

[B33] Nguyen A. W. Daugherty P. S. (2005). Evolutionary optimization of fluorescent proteins for intracellular FRET. *Nat. Biotechnol.* 23 355–360. 10.1038/nbt1066 15696158

[B34] Nollmann F. I. Dauth C. Mulley G. Kegler C. Kaiser M. Waterfield N. R. (2015a). Insect-specific production of new GameXPeptides in *Photorhabdus luminescens* TTO1, widespread natural products in entomopathogenic bacteria. *Chembiochem* 16 205–208. 10.1002/cbic.201402603 25425189

[B35] Nollmann F. I. Heinrich A. K. Brachmann A. O. Morisseau C. Mukherjee K. Casanova-Torres ÁM. (2015b). A *Photorhabdus* natural product inhibits insect juvenile hormone epoxide hydrolase. *Chembiochem* 16 766–771. 10.1002/cbic.201402650 25711603 PMC4486325

[B36] Ohlendorf B. Simon S. Wiese J. Imhoff J. F. (2011). Szentiamide, an N-formylated cyclic depsipeptide from *Xenorhabdus szentirmaii* DSM 16338T. *Nat. Prod. Commun.* 6 1247–1250. 21941889

[B37] Pareja E. Pareja-Tobes P. Manrique M. Pareja-Tobes E. Bonal J. Tobes R. (2006). ExtraTrain: a database of extragenic regions and transcriptional information in prokaryotic organisms. *BMC Microbiol.* 6:29 10.1186/1471-2180-6-29 PMC1453763 16539733

[B38] Reimer D. Cowles K. N. Proschak A. Nollmann F. I. Dowling A. J. Kaiser M. (2013). Rhabdopeptides as insect-specific virulence factors from entomopathogenic bacteria. *Chembiochem* 14 1991–1997. 10.1002/cbic.201300205 24038745

[B39] Reimer D. Nollmann F. I. Schultz K. Kaiser M. Bode H. B. (2014). Xenortide Biosynthesis by Entomopathogenic *Xenorhabdus nematophila*. *J. Nat. Prod.* 77 1976–1980. 10.1021/np500390b 25080196

[B40] Schimming O. Fleischhacker F. Nollmann F. I. Bode H. B. (2014). Yeast homologous recombination cloning leading to the novel peptides ambactin and xenolindicin. *Chembiochem* 15 1290–1294. 10.1002/cbic.201402065 24816640

[B41] Shimada T. Yamamoto K. Ishihama A. (2009). Involvement of the leucine response transcription factor LeuO in regulation of the genes for sulfa drug efflux. *J. Bacteriol.* 191 4562–4571. 10.1128/JB.00108-09 19429622 PMC2704711

[B42] Tani T. H. Khodursky A. Blumenthal R. M. Brown P. O. Matthews R. G. (2002). Adaptation to famine: a family of stationary-phase genes revealed by microarray analysis. *Proc. Natl. Acad. Sci. U.S.A.* 99 13471–13476. 10.1073/pnas.212510999 12374860 PMC129697

[B43] Thomas G. M. Poinar G. O. (1979). *Xenorhabdus* gen. nov., a genus of entomopathogenic, nematophilic bacteria of the family enterobacteriaceae. *Int. J. Syst. Bacteriol.* 29 352–360. 10.1099/00207713-29-4-352

[B44] Tobias N. J. Heinrich A. K. Eresmann H. Wright P. R. Neubacher N. Backofen R. (2016). *Photorhabdus*-nematode symbiosis is dependent on hfq-mediated regulation of secondary metabolites. *Environ. Microbiol.* 19 119–129. 10.1111/1462-2920.13502 27555343

[B45] Uliczka F. Pisano F. Kochut A. Opitz W. Herbst K. Stolz T. (2011). Monitoring of gene expression in bacteria during infections using an adaptable set of bioluminescent, fluorescent and colorigenic fusion vectors. *PLoS ONE* 6:e20425 10.1371/journal.pone.0020425 PMC3108616 21673990

[B46] Whitaker W. B. Parent M. A. Boyd A. Richards G. P. Boyd E. F. (2012). The Vibrio parahaemolyticus ToxRS regulator is required for stress tolerance and colonization in a novel orogastric streptomycin-induced adult murine model. *Infect. Immun.* 80 1834–1845. 10.1128/IAI.06284-11 22392925 PMC3347455

[B47] Xu J. Lohrke S. Hurlbert I. M. Hurlbert R. E. (1989). Transformation of *Xenorhabdus nematophilus*. *Appl. Environ. Microbiol.* 55 806–812. 2729982 10.1128/aem.55.4.806-812.1989 PMC184206

[B48] Zhou Y. Choi Y.-L. Sun M. Yu Z. (2008). Novel roles of Bacillus thuringiensis to control plant diseases. *Appl. Microbiol. Biotechnol.* 80 563–572. 10.1007/s00253-008-1610-3 18654770

