# Supplementary material for: The Global Regulators Lrp, LeuO, and HexA Control Secondary Metabolism in Entomopathogenic Bacteria
Source: Front Microbiol. 2017 Feb 17;8:209. doi: 10.3389/fmicb.2017.00209 (PMC5313471; doi:10.3389/fmicb.2017.00209)
Supplement: Supplementary file 1 [file Data_Sheet_1.DOCX]

**Supporting information**

**Plasmids.** Features of pBAD30_*kanR* and pYEYP2.2 are listed in Table S1 and S2. The DNA sequences of the plasmids are depicted below the corresponding tables.

**Table S1:** Features of pBAD30_*kanR*

| **Name** | **Type** | **Start** | **Stop** | **Length (bp)** |
| --- | --- | --- | --- | --- |
| **p15A** | Origin of Replication | 3,955 | 4,782 | 828 |
| ***kanR*** | Gene | 2,605 | 1,788 | 818 |
| **ara01** | Operator | 1,172 | 1,161 | 12 |
| **ara02** | Operator | 1,019 | 1,004 | 16 |
| ***araC*** | Gene | 974 | 96 | 879 |
| ***P_BAD_*** | Promoter | 1,248 | 1,276 | 29 |

pBAD30_*kanR* DNA sequence

1 10 20 30 40 50

| | | | | |

ATCGATGCATAATGTGCCTGTCAAATGGACGAAGCAGGGATTCTGCAAAC

CCTATGCTACTCCGTCAAGCCGTCAATTGTCTGATTCGTTACCAATTATG

ACAACTTGACGGCTACATCATTCACTTTTTCTTCACAACCGGCACGGAAC

TCGCTCGGGCTGGCCCCGGTGCATTTTTTAAATACCCGCGAGAAATAGAG

TTGATCGTCAAAACCAACATTGCGACCGACGGTGGCGATAGGCATCCGGG

TGGTGCTCAAAAGCAGCTTCGCCTGGCTGATACGTTGGTCCTCGCGCCAG

CTTAAGACGCTAATCCCTAACTGCTGGCGGAAAAGATGTGACAGACGCGA

CGGCGACAAGCAAACATGCTGTGCGACGCTGGCGATATCAAAATTGCTGT

CTGCCAGGTGATCGCTGATGTACTGACAAGCCTCGCGTACCCGATTATCC

ATCGGTGGATGGAGCGACTCGTTAATCGCTTCCATGCGCCGCAGTAACAA

TTGCTCAAGCAGATTTATCGCCAGCAGCTCCGAATAGCGCCCTTCCCCTT

GCCCGGCGTTAATGATTTGCCCAAACAGGTCGCTGAAATGCGGCTGGTGC

GCTTCATCCGGGCGAAAGAACCCCGTATTGGCAAATATTGACGGCCAGTT

AAGCCATTCATGCCAGTAGGCGCGCGGACGAAAGTAAACCCACTGGTGAT

ACCATTCGCGAGCCTCCGGATGACGACCGTAGTGATGAATCTCTCCTGGC

GGGAACAGCAAAATATCACCCGGTCGGCAAACAAATTCTCGTCCCTGATT

TTTCACCACCCCCTGACCGCGAATGGTGAGATTGAGAATATAACCTTTCA

TTCCCAGCGGTCGGTCGATAAAAAAATCGAGATAACCGTTGGCCTCAATC

GGCGTTAAACCCGCCACCAGATGGGCATTAAACGAGTATCCCGGCAGCAG

GGGATCATTTTGCGCTTCAGCCATACTTTTCATACTCCCGCCATTCAGAG

AAGAAACCAATTGTCCATATTGCATCAGACATTGCCGTCACTGCGTCTTT

TACTGGCTCTTCTCGCTAACCAAACCGGTAACCCCGCTTATTAAAAGCAT

TCTGTAACAAAGCGGGACCAAAGCCATGACAAAAACGCGTAACAAAAGTG

TCTATAATCACGGCAGAAAAGTCCACATTGATTATTTGCACGGCGTCACA

CTTTGCTATGCCATAGCATTTTTATCCATAAGATTAGCGGATCCTACCTG

ACGCTTTTTATCGCAACTCTCTACTGTTTCTCCATACCCGTTTTTTTGGG

CTAGCGAATTCGAGCTCGGTACCCGGGGATCCTCTAGAGTCGACCTGCAG

GCATGCAAGCTTGGCTGTTTTGGCGGATGAGAGAAGATTTTCAGCCTGAT

ACAGATTAAATCAGAACGCAGAAGCGGTCTGATAAAACAGAATTTGCCTG

GCGGCAGTAGCGCGGTGGTCCCACCTGACCCCATGCCGAACTCAGAAGTG

AAACGCCGTAGCGCCGATGGTAGTGTGGGGTCTCCCCATGCGAGAGTAGG

GAACTGCCAGGCATCAAATAAAACGAAAGGCTCAGTCGAAAGACTGGGCC

TTTCGTTTTATCTGTTGTTTGTCGGTGAACGCTCTCCTGAGTAGGACAAA

TCCGCCGGGAGCGGATTTGAACGTTGCGAAGCAACGGCCCGGAGGGTGGC

GGGCAGGACGCCCGCCATAAACTGCCAGGCATCAAATTAAGCAGAACTCG

AGCCATGGAACTTGGTCTGACAGTTACCAATGCTTAATCTTAGAAAAACT

CATCGAGCATCAAATGAAACTGCAATTTATTCATATCAGGATTATCAATA

CCATATTTTTGAAAAAGCCGTTTCTGTAATGAAGGAGAAAACTCACCGAG

GCAGTTCCATAGGATGGCAAGATCCTGGTATCGGTCTGCGATTCCGACTC

GTCCAACATCAATACAACCTATTAATTTCCCCTCGTCAAAAATAAGGTTA

TCAAGTGAGAAATCACCATGAGTGACGACTGAATCCGGTGAGAATGGCAA

AAGTTTATGCATTTCTTTCCAGACTTGTTCAACAGGCCAGCCATTACGCT

CGTCATCAAAATCACTCGCATCAACCAAACCGTTATTCATTCGTGATTGC

GCCTGAGCGAGACGAAATACGCGGTCGCTGTTAAAAGGACAATTACAAAC

AGGAATCGAATGCAACCGGCGCAGGAACACTGCCAGCGCATCAACAATAT

TTTCACCTGAATCAGGATATTCTTCTAATACCTGGAATGCTGTTTTCCCG

GGGATCGCAGTGGTGAGTAACCATGCATCATCAGGAGTACGGATAAAATG

CTTGATGGTCGGAAGAGGCATAAATTCCGTCAGCCAGTTTAGTCTGACCA

TCTCATCTGTAACATCATTGGCAACGCTACCTTTGCCATGTTTCAGAAAC

AACTCTGGCGCATCGGGCTTCCCATACAATCGATAGATTGTCGCACCTGA

TTGCCCGACATTATCGCGAGCCCATTTATACCCATATAAATCAGCATCCA

TGTTGGAATTTAATCGCGGCCTAGAGCAAGACGTTTCCCGTTGAATATGG

CTCATACTCTTCCTTTTTCAATATTATTGAAGCATTTATCAGGGTTATTG

TCTCATGAGCGGATACATATTTGAATGTATTTAGAAAAATAAACAAATAG

GGGTTCCGCGCACATTTCCCCGAAAAGTGCCACCTGACGTCTAAGAAACC

ATTATTATCCTCGAGCCATGGCATATATACTTTAGATTGATTTACGCGCC

CTGTAGCGGCGCATTAAGCGCGGCGGGTGTGGTGGTTACGCGCAGCGTGA

CCGCTACACTTGCCAGCGCCCTAGCGCCCGCTCCTTTCGCTTTCTTCCCT

TCCTTTCTCGCCACGTTCGCCGGCTTTCCCCGTCAAGCTCTAAATCGGGG

GCTCCCTTTAGGGTTCCGATTTAGTGCTTTACGGCACCTCGACCCCAAAA

AACTTGATTTGGGTGATGGTTCACGTAGTGGGCCATCGCCCTGATAGACG

GTTTTTCGCCCTTTGACGTTGGAGTCCACGTTCTTTAATAGTGGACTCTT

GTTCCAAACTTGAACAACACTCAACCCTATCTCGGGCTATTCTTTTGATT

TATAAGGGATTTTGCCGATTTCGGCCTATTGGTTAAAAAATGAGCTGATT

TAACAAAAATTTAACGCGAATTTTAACAAAATATTAACGTTTACAATTTA

AAAGGATCTAGGTGAAGATCCTTTTTGATAATCTCATGACCAAAATCCCT

TAACGTGAGTTTTCGTTCCACTGAGCGTCAGACCCCGTAGAAAAGATCAA

AGGATCTTCTTGAGATCCTTTTTTTCTGCGCGTAATCTGCTGCTTGCAAA

CAAAAAAACCACCGCTACCAGCGGTGGTTTGTTTGCCGGATCAAGAGCTA

CCAACTCTTTTTCCGAAGGTAACTGGCTTCAGCAGAGCGCAGATACCAAA

TACTGTCCTTCTAGTGTAGCCGTAGTTAGGCCACCACTTCAAGAACTCTG

TAGCACCGCCTACATACCTCGCTCTGCTAATCCTGTTACCAGTCAGGCAT

TTGAGAAGCACACGGTCACACTGCTTCCGGTAGTCAATAAACCGGTAAAC

CAGCAATAGACATAAGCGGCTATTTAACGACCCTGCCCTGAACCGACGAC

CGGGTCGAATTTGCTTTCGAATTTCTGCCATTCATCCGCTTATTATCACT

TATTCAGGCGTAGCACCAGGCGTTTAAGGGCACCAATAACTGCCTTAAAA

AAATTACGCCCCGCCCTGCCACTCATCGCAGTACTGTTGTAATTCATTAA

GCATTCTGCCGACATGGAAGCCATCACAGACGGCATGATGAACCTGAATC

GCCAGCGGCATCAGCACCTTGTCGCCTTGCGTATAATATTTGCCGCTAGC

GGAGTGTATACTGGCTTACTATGTTGGCACTGATGAGGGTGTCAGTGAAG

TGCTTCATGTGGCAGGAGAAAAAAGGCTGCACCGGTGCGTCAGCAGAATA

TGTGATACAGGATATATTCCGCTTCCTCGCTCACTGACTCGCTACGCTCG

GTCGTTCGACTGCGGCGAGCGGAAATGGCTTACGAACGGGGCGGAGATTT

CCTGGAAGATGCCAGGAAGATACTTAACAGGGAAGTGAGAGGGCCGCGGC

AAAGCCGTTTTTCCATAGGCTCCGCCCCCCTGACAAGCATCACGAAATCT

GACGCTCAAATCAGTGGTGGCGAAACCCGACAGGACTATAAAGATACCAG

GCGTTTCCCCCTGGCGGCTCCCTCGTGCGCTCTCCTGTTCCTGCCTTTCG

GTTTACCGGTGTCATTCCGCTGTTATGGCCGCGTTTGTCTCATTCCACGC

CTGACACTCAGTTCCGGGTAGGCAGTTCGCTCCAAGCTGGACTGTATGCA

CGAACCCCCCGTTCAGTCCGACCGCTGCGCCTTATCCGGTAACTATCGTC

TTGAGTCCAACCCGGAAAGACATGCAAAAGCACCACTGGCAGCAGCCACT

GGTAATTGATTTAGAGGAGTTAGTCTTGAAGTCATGCGCCGGTTAAGGCT

AAACTGAAAGGACAAGTTTTGGTGACTGCGCTCCTCCAAGCCAGTTACCT

CGGTTCAAAGAGTTGGTAGCTCAGAGAACCTTCGAAAAACCGCCCTGCAA

GGCGGTTTTTTCGTTTTCAGAGCAAGAGATTACGCGCAGACCAAAACGAT

CTCAAGAAGATCATCTTATTAATCAGATAAAATATTTGCTCATGAGCCCG

AAGTGGCGAGCCCGATCTTCCCCATCGGTGATGTCGGCGATATAGGCGCC

AGCAACCGCACCTGTGGCGCCGGTGATGCCGGCCACGATGCGTCCGGCGT

AGAGGATCTGCTCATGTTTGACAGCTTATC

**Table S2:** Features of pYEYP2.2

| **Name** | **Type** | **Start** | **Stop** | **Length (bp)** |
| --- | --- | --- | --- | --- |
| ***kanR*** | Gene | 3,656 | 2,862 | 795 |
| **pSC101*** | Origin of Replication | 1,028 | 2,723 | 1,696 |
| ***ypet*** | Gene | 50 | 766 | 717 |
| **SD** | Shine Dalgarno sequence | 36 | 41 | 6 |

pYEYP2.2 DNA sequence

1 10 20 30 40 50

| | | | | |

CTCGAGGATCCCGGGTACCTGCAGCTAGCGTCGACAGGAGAAATTAAGCA

TGAGCAAAGGTGAAGAACTGTTTACCGGTGTTGTTCCGATTCTGGTTGAA

CTGGATGGTGATGTTAATGGCCACAAATTTTCAGTTAGCGGTGAAGGCGA

AGGTGATGCAACCTATGGTAAACTGACCCTGAAACTGCTGTGTACCACCG

GCAAACTGCCGGTTCCGTGGCCGACCCTGGTTACCACCCTGGGTTATGGT

GTTCAGTGTTTTGCACGTTATCCGGATCATATGAAACAGCACGATTTTTT

CAAAAGCGCAATGCCGGAAGGTTATGTTCAAGAACGTACCATCTTCTTCA

AAGATGACGGCAACTATAAAACCCGTGCCGAAGTTAAATTTGAAGGTGAT

ACCCTGGTGAATCGCATTGAACTGAAAGGCATCGATTTTAAAGAGGATGG

TAATATCCTGGGCCACAAACTGGAATATAATTATAACAGCCACAACGTGT

ATATCACCGCAGACAAACAGAAAAATGGCATCAAAGCCAACTTCAAAATC

CGCCATAATATTGAAGATGGTGGTGTGCAGCTGGCAGATCATTATCAGCA

GAATACCCCGATTGGTGATGGTCCGGTTCTGCTGCCGGATAATCATTATC

TGAGCTATCAGAGCGCACTGTTTAAAGATCCGAATGAAAAACGTGATCAT

ATGGTGCTGCTGGAATTTCTGACCGCAGCAGGTATTACCGAAGGTATGAA

TGAACTGTATAAATGAGCGGCCGCTCTAGAGGCATCAAATAAAACGAAAG

GCTCAGTCGAAAGACTGGGCCTTTCGTTTTATCTGTTGTTTGTCGGTGAA

CGCTCTCCTGAGTAGGACAAATCCGCCGCCCTAGACCTAGGCGTTCGGCT

GCGGCGAGCGGTATCAGCTCACTCAAAGGCGGTAATACGGTTATCCACAG

AATCAGGGGATAACGCAGGAAAGAACATGTGAGCAAAAGGCCAGCAAAAG

GCCAGGAACCGTAAAAAGGCCGCGTTGTCAGATCCTTCCGTATTTAGCCA

GTATGTTCTCTAGTGTGGTTCGTTGTTTTTGCGTGAGCCATGAGAACGAA

CCATTGAGATCATGCTTACTTTGCATGTCACTCAAAAATTTTGCCTCAAA

ACTGGTGAGCTGAATTTTTGCAGTTAAAGCATCGTGTAGTGTTTTTCTTA

GTCCGTTACGTAGGTAGGAATCTGATGTAATGGTTGTTGGTATTTTGTCA

CCATTCATTTTTATCTGGTTGTTCTCAAGTTCGGTTACGAGATCCATTTG

TCTATCTAGTTCAACTTGGAAAATCAACGTATCAGTCGGGCGGCCTCGCT

TATCAACCACCAATTTCATATTGCTGTAAGTGTTTAAATCTTTACTTATT

GGTTTCAAAACCCATTGGTTAAGCCTTTTAAACTCATGGTAGTTATTTTC

AAGCATTAACATGAACTTAAATTCATCAAGGCTAATCTCTATATTTGCCT

TGTGAGTTTTCTTTTGTGTTAGTTCTTTTAATAACCACTCATAAATCCTC

ATAGAGTATTTGTTTTCAAAAGACTTAACATGTTCCAGATTATATTTTAT

GAATTTTTTTAACTGGAAAAGATAAGGCAATATCTCTTCACTAAAAACTA

ATTCTAATTTTTCGCTTGAGAACTTGGCATAGTTTGTCCACTGGAAAATC

TCAAAGCCTTTAACCAAAGGATTCCTGATTTCCACAGTTCTCGTCATCAG

CTCTCTGGTTGCTTTAGCTAATACACCATAAGCATTTTCCCTACTGATGT

TCATCATCTGAGCGTATTGGTTATAAGTGAACGATACCGTCCGTTCTTTC

CTTGTAGGGTTTTCAATCGTGGGGTTGAGTAGTGCCACACAGCATAAAAT

TAGCTTGGTTTCATGCTCCGTTAAGTCATAGCGACTAATCGCTAGTTCAT

TTGCTTTGAAAACAACTAATTCAGACATACATCTCAATTGGTCTAGGTGA

TTTTAATCACTATACCAATTGAGATGGGCTAGTCAATGATAATTACTAGT

CCTTTTCCCGGGAGATCTGGGTATCTGTAAATTCTGCTAGACCTTTGCTG

GAAAACTTGTAAATTCTGCTAGACCCTCTGTAAATTCCGCTAGACCTTTG

TGTGTTTTTTTTGTTTATATTCAAGTGGTTATAATTTATAGAATAAAGAA

AGAATAAAAAAAGATAAAAAGAATAGATCCCAGCCCTGTGTATAACTCAC

TACTTTAGTCAGTTCCGCAGTATTACAAAAGGATGTCGCAAACGCTGTTT

GCTCCTCTACAAAACAGACCTTAAAACCCTAAAGGCTTAAGTAGCACCCT

CGCAAGCTCGGGCAAATCGCTGAATATTCCTTTTGTCTCCGACCATCAGG

CACCTGAGTCGCTGTCTTTTTCGTGACATTCAGTTCGCTGCGCTCACGGC

TCTGGCAGTGAATGGGGGTAAATGGCACTACAGGCGCCTTTTATGGATTC

ATGCAAGGAAACTACCCATAATACAAGAAAAGCCCGTCACGGGCTTCTCA

GGGCGTTTTATGGCGGGTCTGCTATGTGGTGCTATCTGACTTTTTGCTGT

TCAGCAGTTCCTGCCCTCTGATTTTCCAGTCTGACCACTTCGGATTATCC

CGTGACAGGTCATTCAGACTGGCTAATGCACCCAGTAAGGCAGCGGTATC

ATCAACAGGCTTACCCGTCTTACGACTAGTGCTTGGATTCTCACCAATAA

AAAACGCCCGGCGGCAACCGAGCGTTCTGAACAAATCCAGATGGAGTTCT

GAGGTCATTACTGGATCTATCAACAGGAGTCCAAGCGAGCTCTCGAACCC

CAGAGTCCCGCTCAGAAGAACTCGTCAAGAAGGCGATAGAAGGCGATGCG

CTGCGAATCGGGAGCGGCGATACCGTAAAGCACGAGGAAGCGGTCAGCCC

ATTCGCCGCCAAGCTCTTCAGCAATATCACGGGTAGCCAACGCTATGTCC

TGATAGCGGTCCGCCACACCCAGCCGGCCACAGTCGATGAATCCAGAAAA

GCGGCCATTTTCCACCATGATATTCGGCAAGCAGGCATCGCCATGGGTCA

CGACGAGATCCTCGCCGTCGGGCATGCGCGCCTTGAGCCTGGCGAACAGT

TCGGCTGGCGCGAGCCCCTGATGCTCTTCGTCCAGATCATCCTGATCGAC

AAGACCGGCTTCCATCCGAGTACGTGCTCGCTCGATGCGATGTTTCGCTT

GGTGGTCGAATGGGCAGGTAGCCGGATCAAGCGTATGCAGCCGCCGCATT

GCATCAGCCATGATGGATACTTTCTCGGCAGGAGCAAGGTGAGATGACAG

GAGATCCTGCCCCGGCACTTCGCCCAATAGCAGCCAGTCCCTTCCCGCTT

CAGTGACAACGTCGAGCACAGCTGCGCAAGGAACGCCCGTCGTGGCCAGC

CACGATAGCCGCGCTGCCTCGTCCTGCAGTTCATTCAGGGCACCGGACAG

GTCGGTCTTGACAAAAAGAACCGGGCGCCCCTGCGCTGACAGCCGGAACA

CGGCGGCATCAGAGCAGCCGATTGTCTGTTGTGCCCAGTCATAGCCGAAT

AGCCTCTCCACCCAAGCGGCCGGAGAACCTGCGTGCAATCCATCTTGTTC

AATCATGCGAAACGATCCTCATCCTGTCTCTTGATCAGATCTTGATCCCC

TGCGCCATCAGATCCTTGGCGGCAAGAAAGCCATCCAGTTTACTTTGCAG

GGCTTCCCAACCTTACCAGAGGGCGCCCCAGCTGGCAATTCCGACGTCTA

AGAAACCATTATTATCATGACATTAACCTATAAAAATAGGCGTATCACGA

GGCCCTTTCGTCTTCAC

**Rhabdopeptide fragmentation patterns:** Figure S1 shows a comparison of fragmentation patterns from two known *X. nematophila* rhabdopeptides and two putative rhabdopeptide derivatives from *X. szentirmaii*. The characteristic multiple losses of *m/z* Δ113 that correspond to leucine or N-methylated valine and *m/z* Δ99 that corresponds to a loss of valine (Reimer *et. al.* 2013) are also present in the putative derivatives. The putative rhabdopeptides do not show a loss of phenylethylamine *m/z* Δ121 but a loss of *m/z* Δ220 which has not been elucidated yet. The retention times of the known rhabdopeptides and the putative rhabdopeptides are also similar (Rt 8.6-9.4 min).


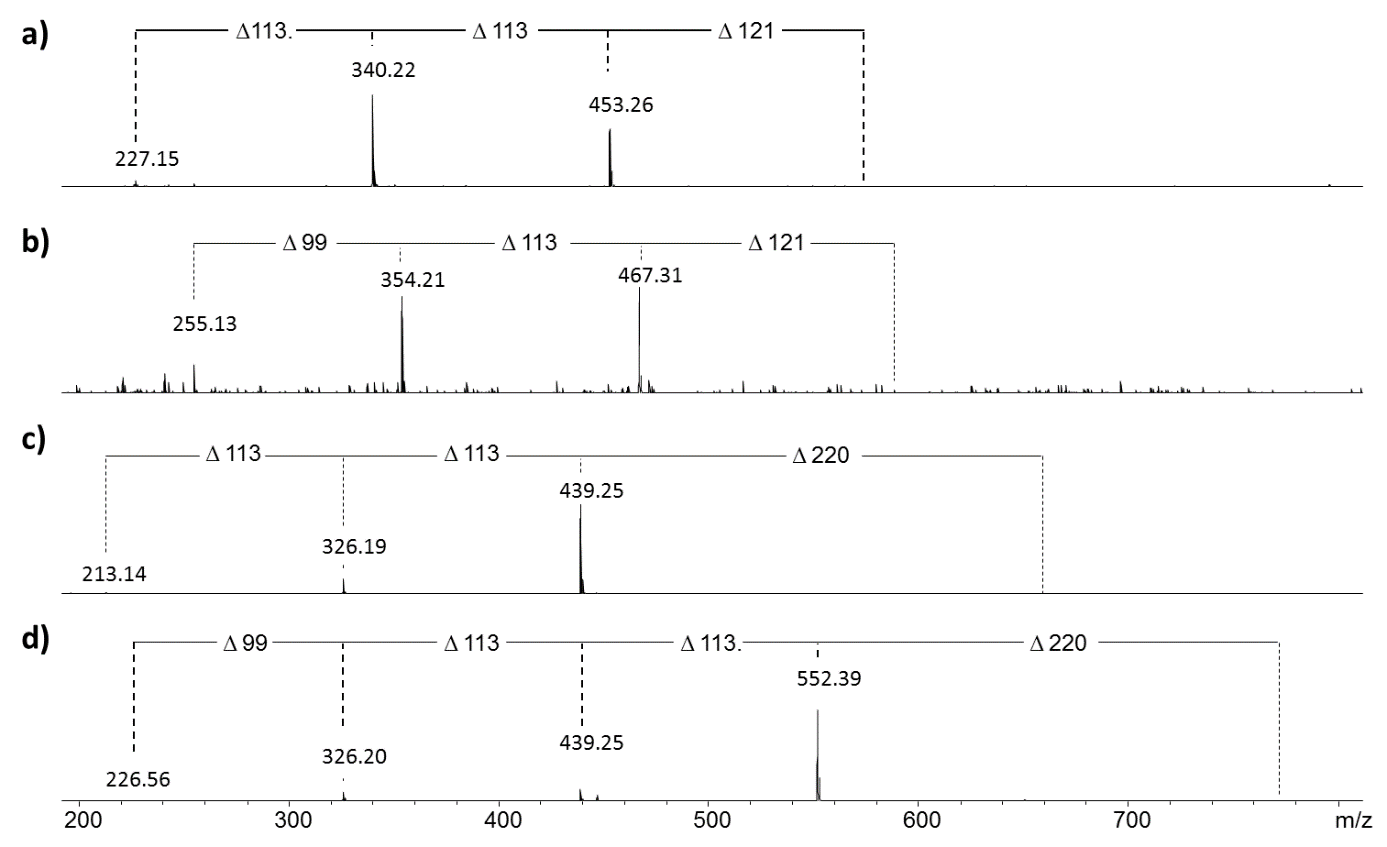


**Figure S1.** a) MS^2^ spectra of rhabdopeptide 1 from *X. nematophila* (Rt: 8.8 min). b) MS^2^ spectra of rhabdopeptide 2 from *X. nematophila* (Rt: 9.4 min). c) MS^2^ spectra of putative rhabdopeptide from *X. szentirmaii* *m/z* 659.3 [M+H]^+^ (Rt: 8.6 min). c) MS^2^ spectra of putative rhabdopeptide from *X. szentirmaii* *m/z* 772.3 [M+H]^+^ (Rt: 9.1 min).

**References**

Reimer, D., Cowles, K. N., Proschak, A., Nollmann, F. I., Dowling, A. J., Kaiser, M., *et al.* (2013). Rhabdopeptides as insect-specific virulence factors from entomopathogenic bacteria. *Chembiochem* 14, 1991–1997. doi: 10.1002/cbic.201300205
